# Pymportx: facilitating next-generation transcriptomics analysis in Python

**DOI:** 10.1093/nargab/lqae160

**Published:** 2024-11-15

**Authors:** Paula Pena González, Dafne Lozano-Paredes, José Luis Rojo-Álvarez, Luis Bote-Curiel, Víctor Javier Sánchez-Arévalo Lobo

**Affiliations:** Molecular Oncology Group, Biosanitary Research Institute, Faculty of Experimental Sciences, Francisco de Vitoria University (UFV), Ctra. Pozuelo-Majadahonda Km. 1800 28223 Pozuelo de Alarcón, Madrid, Spain; Pathology Department, Hospital 12 de Octubre, Av. Córdoba, s/n, 28041 Madrid, Spain; Department of Signal Theory and Communications, Universidad Rey Juan Carlos (URJC), Camino del Molino Nº5. 28942 - Fuenlabrada – Madrid, Spain; Department of Signal Theory and Communications, Universidad Rey Juan Carlos (URJC), Camino del Molino Nº5. 28942 - Fuenlabrada – Madrid, Spain; Department of Signal Theory and Communications, Universidad Rey Juan Carlos (URJC), Camino del Molino Nº5. 28942 - Fuenlabrada – Madrid, Spain; Molecular Oncology Group, Biosanitary Research Institute, Faculty of Experimental Sciences, Francisco de Vitoria University (UFV), Ctra. Pozuelo-Majadahonda Km. 1800 28223 Pozuelo de Alarcón, Madrid, Spain; Pathology Department, Hospital 12 de Octubre, Av. Córdoba, s/n, 28041 Madrid, Spain

## Abstract

The efficient importation of quantified gene expression data is pivotal in transcriptomics. Historically, the R package Tximport addressed this need by enabling seamless data integration from various quantification tools. However, the Python community lacked a corresponding tool, restricting cross-platform bioinformatics interoperability. We introduce Pymportx, a Python adaptation of Tximport, which replicates and extends the original package’s functionalities. Pymportx maintains the integrity and accuracy of gene expression data while improving processing speed and integration within the Python ecosystem. It supports new data formats and includes tools for enhanced data exploration and analysis. Available under the MIT license, Pymportx integrates smoothly with Python’s bioinformatics tools, facilitating a unified and efficient workflow across the R and Python ecosystems. This advancement not only broadens access to Python’s extensive toolset but also fosters interdisciplinary collaboration and the development of cutting-edge bioinformatics analyses.

## Introduction

In the realm of transcriptomics research, the accurate importation and preprocessing of expression data are paramount for reliable biological interpretation. Variations in quantification methodologies, sequencing platforms or experimental setups introduce technical biases that, if unaddressed, can significantly distort the biological conclusions drawn from such data. Numerous strategies have been developed to mitigate these issues, encompassing both classical statistical techniques, such as normalization and batch effect correction, and more recent machine learning approaches. However, many of these methods struggle with the inherent complexities of transcriptomics data, such as low sample sizes or the need to harmonize across multiple batches or studies.

The Tximport tool in R has been pivotal in addressing part of this challenge by providing a robust framework for importing and summarizing transcript quantification data from various sources (1). Its approach effectively bridges the gap between raw sequencing outputs and downstream statistical analysis, but until now a direct counterpart in Python was missing.

Here, we introduce Pymportx, a Python package that faithfully reimplements the Tximport functionality. Pymportx not only allows for the efficient importation of transcriptomics data into Python’s rich data analysis ecosystem but also enhances the process with additional features designed to handle the diverse and complex nature of such data. By facilitating a seamless transition of Tximport’s capabilities into Python, Pymportx opens new avenues for transcriptomics research that leverages Python’s advanced computational libraries.

In direct comparison with the original R package (1), Pymportx demonstrates equivalent functionality in aggregating and preparing transcriptomics data for analysis, ensuring that researchers can rely on the integrity and accuracy of their processed data. Furthermore, Pymportx introduces improvements in processing speed and flexibility, allowing for more efficient workflows and the integration of Python-based data analysis tools. This makes Pymportx not just a tool for data importation but a cornerstone for the next generation of transcriptomics analysis in Python, enabling researchers to tackle the complexities of biological data with unprecedented ease and efficiency.

## Materials and methods

### Software development and implementation

The Pymportx package was developed using Python 3.12.2. We leveraged established libraries such as Pandas version 2.1.1 for data manipulation and NumPy version 1.26.3 for numerical calculations, ensuring high performance and interoperability within the Python ecosystem. The package was structured to mimic the core functionalities of the Tximport R package (release 3.19 in R version 4.2.3), extending them with additional features to accommodate Python’s capabilities and the needs of the bioinformatics community. These new features include outputting results in an AnnData format, which integrates seamlessly with the scverse ecosystem, enhancing interoperability with single-cell and other bioinformatics analyses (2).

### Data sources and formats

For testing and validation, transcript quantification data were sourced from publicly available RNA sequencing (RNA-seq) datasets, which included outputs from tools such as Salmon, Kallisto, Sailfish and RSEM included in the Tximport R package release 3.19.

### Data processing

Quantification data were imported using Pymportx, which parses and aggregates raw count data into a structured Python DataFrame. This process includes normalization steps, which adjust for sequencing depth and other technical variabilities, using methods adapted from the Tximport package.

### Validation and benchmarking

The performance of Pymportx was benchmarked against the original Tximport package by comparing processing speed, data integrity and analysis outcomes. Additionally, the extension of functionalities was validated through case studies, demonstrating the tool’s capability in handling complex transcriptomics data and producing replicable results. All computational analyses were conducted on a high-performance computing system equipped with 1.0 TB of RAM, 128 CPU cores distributed across two sockets with 64 cores per socket. To quantify the similarity of the outputs produced by Pymportx and Tximport, correlation coefficient, mean absolute error (MAE) and mean squared error (MSE) were calculated between the resulting matrices from each test, affirming the equivalence of the outputs. Additionally, the execution time for each package was measured across all tests, with each test repeated 10 times to determine the average runtime and standard deviation.

## Results

Pymportx adapts the core functionality of the R package Tximport for use within the Python ecosystem, focusing primarily on the accurate and efficient importation and summarization of transcript quantification data from RNA-seq experiments. The tool is designed to handle outputs from a variety of transcript quantification software, effectively converting them into a format that is readily usable for downstream analyses, including differential expression analysis with tools such as PyDESeq2 (3).

### Available features and code structure

Pymportx incorporates features that align with the primary capabilities of its R counterpart. The tool is structured around a core set of functions that facilitate these processes, ensuring that users can efficiently transition from data quantification to analysis within the Python environment. This achievement includes the ability to process quantification outputs into summarized count matrices, ensuring compatibility with a range of data formats from leading quantification tools such as Salmon, Kallisto, Sailfish and RSEM. Additionally, Pymportx supports essential transformations and scaling adjustments required for data analysis. These enhancements are integrated through a core set of functions designed to streamline the transition from data quantification to analysis within the Python environment, enhancing usability and efficiency for users.

The architecture of Pymportx leverages widely used Python libraries such as Pandas for data manipulation and NumPy for numerical computations, ensuring high performance and interoperability with the broader Python data science ecosystem. By building on the foundation provided by established Python libraries and adhering to best practices in software development, Pymportx presents a robust and user-friendly tool for the bioinformatics community. Its open-source nature, under the MIT license, invites contributions from developers and researchers alike, fostering a collaborative environment for the continuous improvement and expansion of its functionalities.

In summary, Pymportx is a vital link in the chain of RNA-seq data analysis in Python, enabling researchers to effectively bridge the gap between data quantification and expression analysis. With its focus on accuracy, efficiency and interoperability, Pymportx sets the stage for advanced bioinformatics research conducted entirely within the Python ecosystem.

### Comparison with Tximport

**Figure 1.**
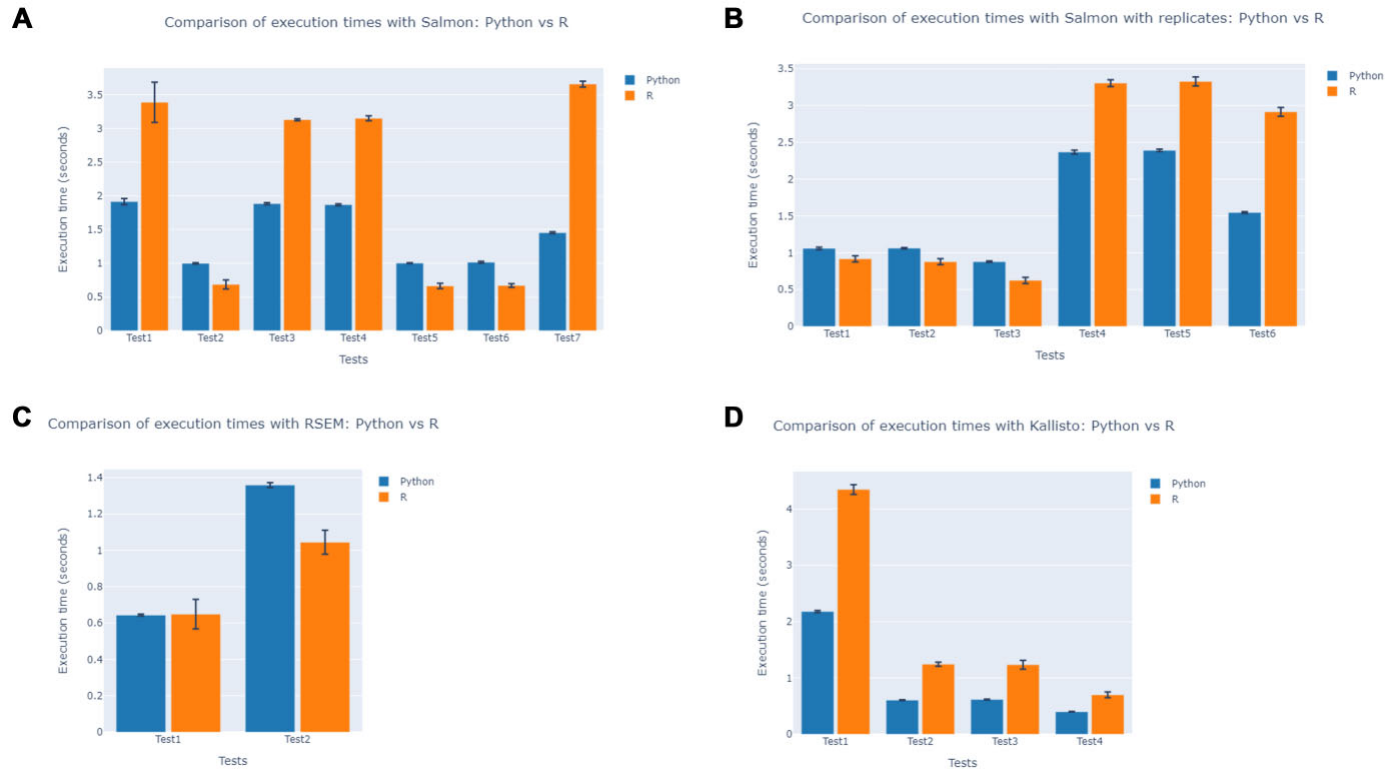
Comparison of computational efficiency between Pymportx and Tximport. This figure shows a comparative analysis of processing speeds between Pymportx (Python implementation) and Tximport (R implementation). Panel (**A**) displays a bar graph with the average computation time for processing transcript quantification data from Salmon output files. Panel (**B**) shows the average computation time for Salmon output files with replicates. Panel (**C**) presents results for RSEM output files, while panel (**D**) focuses on Kallisto output files. Each data point represents the mean computation time, with error bars indicating standard deviations. Statistical significance is marked by asterisks (***P< 0.001; **P< 0.01; *P< 0.05; n.s. = not significant), underscoring the differences in processing speeds between the two packages across various datasets.

To evaluate the performance and accuracy of Pymportx, comparative tests were conducted using tests from the Tximport GitHub repository (https://github.com/thelovelab/tximport/tree/devel/tests). These tests cover a range of input scenarios, utilizing output files from three popular RNA-seq quantification tools, namely Salmon, RSEM and Kallisto, in which each test is structured with its own set of input files and specific parameters. The resulting matrices from these tests are the abundance, length and count matrices, where the abundance matrix represents the estimated abundance of each transcript or gene in transcripts per million, the length matrix contains the effective lengths of transcripts or genes, and the count matrix provides the estimated read counts for each feature. These tests were executed on the same machine using both Tximport and Pymportx. The results are presented in Tables 1–4, demonstrating that outputs from both packages are essentially identical, confirming the accuracy of the Pymportx implementation. Any slight differences observed between the outputs are attributed to inherent limitations of floating-point arithmetic and rounding errors in numerical computations. To quantify this similarity, correlation coefficient, MAE and MSE were calculated between the resulting matrices from each test, all of which support the equivalence of the outputs. Furthermore, the execution time for each package was measured across all tests, with each test repeated 10 times to calculate the average runtime and standard deviation. All measurements were conducted on the same computer for both R and Python implementations to ensure a fair comparison. The results are presented in Figure 1, providing a comprehensive comparison of the computational efficiency of both packages. In addition, to assess the statistical significance of the differences in execution times, a two-tailed Welch’s *t*-test was performed for each test case with 10 degrees of freedom.

**Table 1.** Matrices resulting from the tests for the Salmon output files

Test	Matrix	Correlation	MAE	MSE
Test 1	Abundance	1.0	5.055e−16	3.929e−28
Test 1	Count	1.0	1.581e−14	1.466e−25
Test 1	Length	1.0	6.501e−13	2.797e−24
Test 2	Abundance	1.0	0.0	0.0
Test 2	Count	1.0	0.0	0.0
Test 2	Length	1.0	0.0	0.0
Test 3	Abundance	1.0	5.055e−16	3.929e−28
Test 3	Count	1.0	4.175e−13	5.393e−23
Test 3	Length	1.0	6.501e−13	2.797e−24
Test 4	Abundance	1.0	5.055e−16	3.929e−28
Test 4	Count	1.0	4.522e−13	3.229e−23
Test 4	Length	1.0	6.501e−13	2.797e−24
Test 5	Abundance	1.0	0.0	0.0
Test 5	Count	1.0	1.234e−13	1.402e−23
Test 5	Length	1.0	0.0	0.0
Test 6	Abundance	1.0	0.0	0.0
Test 6	Count	1.0	1.300e−13	7.912e−24
Test 6	Length	1.0	0.0	0.0
Test 7	Abundance	1.0	0.0	0.0
Test 7	Count	1.0	1.281e−13	1.177e−23
Test 7	Length	1.0	0.0	0.0

**Table 2.** Matrices resulting from the tests for the Salmon output files with replicates

Test	Matrix	Correlation	MAE	MSE
Test 1	Abundance	1.0	0.0	0.0
Test 1	Count	1.0	0.0	0.0
Test 1	Length	1.0	0.0	0.0
Test 2	Abundance	1.0	0.0	0.0
Test 2	Count	1.0	0.0	0.0
Test 2	Length	1.0	0.0	0.0
Test 3	Abundance	1.0	0.0	0.0
Test 3	Count	1.0	0.0	0.0
Test 3	Length	1.0	0.0	0.0
Test 4	Abundance	1.0	8.375e−16	6.587e−28
Test 4	Count	1.0	2.676e−14	5.122e−25
Test 4	Length	1.0	1.109e−12	4.918e−24
Test 5	Abundance	1.0	8.375e−16	6.587e−28
Test 5	Count	1.0	2.676e−14	5.122e−25
Test 5	Length	1.0	1.109e−12	4.918e−24
Test 6	Abundance	1.0	8.375e−16	6.587e−28
Test 6	Count	1.0	2.676e−14	5.122e−25
Test 6	Length	1.0	1.109e−12	4.918e−24

**Table 3.** Matrices resulting from the tests for the RSEM output files

Test	Matrix	Correlation	MAE	MSE
Test 1	Abundance	1.0	0.0	0.0
Test 1	Count	1.0	0.0	0.0
Test 1	Length	1.0	0.0	0.0
Test 2	Abundance	1.0	0.0	0.0
Test 2	Count	1.0	0.0	0.0
Test 2	Length	1.0	0.033	0.033

**Table 4.** Matrices resulting from the tests for the Kallisto output files

Test	Matrix	Correlation	MAE	MSE
Test 1	Abundance	1.0	0.0	0.0
Test 1	Count	1.0	0.0	0.0
Test 1	Length	1.0	0.0	0.0
Test 2	Abundance	1.0	0.0	0.0
Test 2	Count	1.0	0.0	0.0
Test 2	Length	1.0	0.0	0.0
Test 3	Abundance	1.0	0.0	0.0
Test 3	Count	1.0	0.0	0.0
Test 3	Length	1.0	0.0	0.0
Test 4	Abundance	1.0	0.0	0.0
Test 4	Count	1.0	0.0	0.0
Test 4	Length	1.0	0.0	0.0

This thorough comparison provides a robust assessment of Pymportx accuracy and performance relative to its R counterpart, offering valuable insights into its efficacy as a Python-based alternative for transcript-level data analysis. The combination of output validation and performance benchmarking demonstrates the reliability and potential advantages of Pymportx in the field of bioinformatics data processing.

## Discussion

In this paper, we introduced Pymportx, a Python library that serves as a direct analog to the R package Tximport, designed for the streamlined importation and summarization of transcriptomics data (1). This tool marks a significant step toward creating a cohesive ecosystem for transcriptomics analysis in Python. Notably, Pymportx facilitates a seamless linkage with PyDESeq2 (3), a Python adaptation of the R package DESeq2 (1,4) which is renowned for its capabilities in differential gene expression analysis. This integration is pivotal for researchers seeking to maintain a continuous workflow entirely within Python, especially those utilizing quantification outputs from tools such as Salmon and Kallisto (5).

The advent of Pymportx extends far beyond mere data importation; it is about enabling researchers to harness the full power of Python for transcriptomics analysis. By ensuring compatibility with PyDESeq2, we bridge the gap between data preprocessing and differential expression analysis, allowing for a streamlined, efficient workflow that leverages Python’s computational prowess and its rich ecosystem of data analysis tools. This continuity is critical for facilitating sophisticated analyses without the need to oscillate between programming languages, thus optimizing both the analytical process and the utilization of computational resources.

Pymportx shares similarities with Pytximport (https://github.com/complextissue/pytximport) but introduces key differences to improve usability. While both support AnnData outputs, Pymportx takes a modular approach to input types, offering separate functions to simplify the interface and prevent misuse. Both packages support count calculations from abundance and allow switching between transcript- and gene-level expression, as well as handling inferential replicates. However, Pymportx aligns more closely with the R ecosystem, using terminology such as dropInfReps, varReduce and infRepStat, which will feel familiar to users transitioning from R-based workflows. This consistency in terminology ensures a smoother adaptation for users coming from R, maintaining alignment with the tools they are already accustomed to.

The open-source nature of Pymportx, under the MIT license, underscores our vision of collaborative advancement in the bioinformatics field. We encourage the community’s engagement with Pymportx, aiming for enhancements, extensions and innovations that will drive the tool forward. This collaborative spirit is integral to the development of a robust, dynamic ecosystem of bioinformatics tools in Python.

Python’s ascendancy as a general-purpose programming language, coupled with its extensive application in data science, machine learning and other computational fields, positions it as an ideal platform for bioinformatics. Through tools such as Pymportx, we not only facilitate this transition but also enrich the analytical capabilities available to researchers, empowering them to achieve more comprehensive, nuanced biological insights.

In the era of large language models and automated code generation, the development of Pymportx serves as a beacon for the potential to expedite the porting of essential bioinformatics tools to Python, while emphasizing the indispensable role of human expertise in ensuring accuracy and optimizing performance.

As we look forward to the continued evolution of Pymportx, our commitment to open-source principles and community collaboration remains steadfast. By fostering an environment of shared knowledge and collective innovation, we anticipate that Pymportx will play a crucial role in advancing the Python bioinformatics ecosystem, paving the way for a future where complex, multistage analyses are conducted with unprecedented efficiency and precision.

## Data Availability

Pymportx is released as an open-source software under the MIT license. The source code is available on GitHub at https://github.com/victorsanchezarevalo/Pymportx (permanent DOI: https://doi.org/10.6084/m9.figshare.27324132).
